# Development and Verify of Survival Analysis Models for Chinese Patients With Systemic Lupus Erythematosus

**DOI:** 10.3389/fimmu.2022.900332

**Published:** 2022-06-24

**Authors:** Linyu Geng, Wenqiang Qu, Jun Liang, Wei Kong, Xue Xu, Wenyou Pan, Lin Liu, Min Wu, Fuwan Ding, Huaixia Hu, Xiang Ding, Hua Wei, Yaohong Zou, Xian Qian, Meimei Wang, Jian Wu, Juan Tao, Jun Tan, Zhanyun Da, Miaojia Zhang, Jing Li, Huayong Zhang, Xuebing Feng, Jiaqi Chen, Lingyun Sun

**Affiliations:** ^1^ Department of Rheumatology and Immunology, The Affiliated Drum Tower Hospital of Nanjing University Medical School, Nanjing, China; ^2^ School of Computer and Information, Hohai University, Nanjing, China; ^3^ Department of Rheumatology, Huai’an First People’s Hospital, Huai’an, China; ^4^ Department of Rheumatology, Xuzhou Central Hospital, Xuzhou, China; ^5^ Department of Rheumatology, The Third Affiliated Hospital of Soochow University, Changzhou, China; ^6^ Department of Endocrinology, Yancheng Third People’s Hospital, Yancheng, China; ^7^ Department of Rheumatology, The Second People’s Hospital of Lianyungang, Lianyungang, China; ^8^ Department of Rheumatology, The First People’s Hospital of Lianyungang, Lianyungang, China; ^9^ Department of Rheumatology, Northern Jiangsu People’s Hospital, Yangzhou, China; ^10^ Department of Rheumatology, Wuxi People’s Hospital, Wuxi, China; ^11^ Department of Rheumatology, Jiangsu Province Hospital of Traditional Chinese Medicine, Nanjing, China; ^12^ Department of Rheumatology, Southeast University Zhongda Hospital, Nanjing, China; ^13^ Department of Rheumatology, The First Affiliated Hospital of Soochow University, Suzhou, China; ^14^ Department of Rheumatology, Wuxi TCM Hospital, Wuxi, China; ^15^ Department of Rheumatology, Zhenjiang First People’s Hospital, Zhenjiang, China; ^16^ Department of Rheumatology, Affiliated Hospital of Nantong University, Nantong, China; ^17^ Department of Rheumatology, Jiangsu Province Hospital, Nanjing, China; ^18^ Department of Rheumatology, Affiliated Hospital of Jiangsu University, Zhenjiang, China

**Keywords:** systemic lupus erythematosus, survival analysis, neural network, regression model, data mining

## Abstract

**Background:**

The aim of this study is to develop survival analysis models of hospitalized systemic lupus erythematosus (h-SLE) patients in Jiangsu province using data mining techniques to predict patient survival outcomes and survival status.

**Methods:**

In this study, based on 1999–2009 survival data of 2453 hospitalized SLE (h-SLE) patients in Jiangsu Province, we not only used the Cox proportional hazards model to analyze patients’ survival factors, but also used neural network models to predict survival outcomes. We used semi-supervised learning to label the censored data and introduced cost-sensitivity to achieve data augmentation, addressing category imbalance and pseudo label credibility. In addition, the risk score model was developed by logistic regression.

**Results:**

The overall accuracy of the survival outcome prediction model exceeded 0.7, and the sensitivity was close to 0.8, and through the comparative analysis of multiple indicators, our model outperformed traditional classifiers. The developed survival risk assessment model based on logistic regression found that there was a clear threshold, i.e., a survival threshold indicating the survival risk of patients, and cardiopulmonary and neuropsychiatric involvement, abnormal blood urea nitrogen levels and alanine aminotransferase level had the greatest impact on patient survival time. In addition, the study developed a graphical user interface (GUI) integrating survival analysis models to assist physicians in diagnosis and treatment.

**Conclusions:**

The proposed survival analysis scheme identifies disease-related pathogenic and prognosis factors, and has the potential to improve the effectiveness of clinical interventions.

## Introduction

Systemic lupus erythematosus (SLE) is a chronic systemic autoimmune disease with significant morbidity and mortality (1, 2). In fact, 1 in 30 hospitalizations culminates in death, and approximately 10% die within 5 years of onset (3–5), the treatment dilemma lies in the lack of precisely targeted treatment (6). Previously, blood pressure, serum complement levels, anti–double-stranded DNA (anti-dsDNA) antibody levels, urine sediment, urine protein-to-creatinine ratio, and surrogates of renal function, etc., were used to monitor mortality, organ involvement, and treatment response in SLE. However, current single clinical indicators, and even predictive models lack sufficient sensitivity and specificity to accurately predict mortality, organ involvement and subsequent precise treatment, thus requiring the development of a new accurate and reliable system for clinical applications.

Most previous survival analyses have focused on the quantitative analysis of survival factors, survival time or rate (7–9). Blanco et al., used univariate analysis and multivariate Cox proportional risk regression analysis to calculate survival probabilities and identify variables associated with survival (10). Similarly, Massardo et al. studied 218 Chilean SLE patients and applied multivariate analysis to identify risk factors affecting survival (11). Kasitanon et al. used the Kaplan-Meier method to estimate the survival probabilities of SLE patients over time since diagnosis and analyzed the predictors of survival in SLE using Cox proportional risk models (12).

With the progression of computer performance, modern artificial neural networks have been shown to outperform traditional logistic regression models in predicting diagnosis, survival, or mortality of diseases, but have mainly focused on cancer (13, 14). Rajimehr et al. used neural networks to predict the incidence of lupus nephritis in SLE patients, with higher sensitivity and accuracy compared with that of logistic regression models and clinician diagnosis (15). Ceccarelli et al. applied recurrent neural networks to predict potential chronic damage in SLE patients (16).

To date, no neural network has been able to successfully predict the survival outcome of individual patients. In this study, we used not only a Cox proportional risk model (Cox model) to analyze patient survival factors, but also a neural network model to predict patient survival outcomes. The predictive model differs from the Cox model, in that it allows for the existence of censored data; therefore, to address the problems posed by missing sample labels, we used semi-supervised learning to label the censored data. We also introduced cost-sensitivity to achieve data augmentation, addressing the issues of category imbalance and pseudo-labeling. In addition, with logistic regression model, we found a survival threshold between survival and mortality risk scores. The entire survival analysis scheme (**Supplementary Figure 1** **)**, was designed to assist physicians in adequately assessing the survival risk and its influencing factors of hospitalized SLE (h-SLE) patients.

## Methods

### Data Collection and Processing

A total of 2453 individuals, who were all hospitalized for the first time, were retrieved from a longitudinal SLE database collected by the Jiangsu Lupus Collaborative Group from 1999 to 2009. The distribution of the year of admission and diagnosis was shown in **Supplementary Figures 2A, B**. Nearly 70% patients were initially diagnosed with SLE (**Supplementary Figure 2C**), and SLEDAI distribution of these patients was shown in **Supplementary Figure 2D**. From this data set, 2444 samples remained after excluding certain samples with many missing values of characteristics, including 1074 lost-follow-up samples and 1370 follow-up samples. Among the follow-up samples, 1137 survival samples and 233 death samples were included. The demographic and clinical characteristics of these patient samples are presented in **Table 1**, **Supplementary Figure 3**.

**Table 1:** Demographic and clinical characteristics of follow-up h-SLE patients.

Variable	Total (N = 1370)	Surviva l (N = 1137)	Death (N = 233)	P value
**Mean Age ± SD (years)**	34.86±12.42	34.21±12.12	37.07±13.92	**0.0104**
**Female-n (%)**	1265(92.34)	1054(92.70)	211(90.56)	0.3248
**Male-n (%)**	105(7.66)	83(7.30)	22(9.44)	0.3248
**SLEDAI on admission**	14.55±8.25	14.04±8.02	17.04±8.89	3.0117
**SLEDAI at discharge**	6.22±7.01	5.69±6.08	8.77±10.04	**0.0006**
**Organ involvements-n (%)**				
**Mucocutaneous**	913(66.64)	767(67.46)	146(62.66)	0.1807
**Neuropsychiatric**	92(6.72)	59(5.19)	33(14.16)	**0.0000**
**Musculoskeletal**	743(54.23)	630(55.41)	113(48.50)	0.0633
**Cardiopulmonary**	282(20.58)	199(17.50)	83(35.62)	**0.0000**
**Gastrointestinal**	69(5.04)	52(4.57)	17(7.30)	0.1172
**Ocular**	9(0.66)	7(0.62)	2(0.86)	0.9782
**Renal**	699(51.02)	551(48.46)	148(63.52)	**0.0000**
**Haematological**	616(44.96)	484(42.57)	132(56.65)	**0.0001**
**Serology-n (%)**				
**Anti-dsDNA positive**	724(52.85)	589(51.80)	135(57.94)	0.1015
**Anti-Sm positive**	411(30.00)	362(31.84)	49(21.03)	**0.0014**
**Anti-cardiolipin positive**	161(11.75)	140(12.31)	21(9.01)	0.1890
**RF positive**	287(20.95)	241(21.20)	47(21.17)	0.7938
**Medications-n (%)**				
**Prednisone**	937(71.02)	806(70.89)	167(71.76)	0.8717
**Prednisolone**	591(43.14)	463(40.72)	128(54.94)	**0.0001**
**Cyclophosphamide**	575(41.97)	489(43.01)	86(36.91)	0.0999
**Hydroxy chloroquine**	476(34.74)	427(37.55)	49(21.03)	**0.0000**

SLEDAI, SLE disease activity index; RF, rheumatoid factor. Data are presented as mean ± SD, n (%), where n is the total number of patients with valid data in each group. Statistical analysis was performed using the Mann-Whitney U-test and the χ2 test. P values less than 0.05 in bold.

We not only selected structured data, such as admission age, but also processed unstructured data such as primary description. For category features, we used one-hot encoding, and for continuous numerical features, we used the maximum and minimum normalization method for processing. The specific variable assignments were shown in **Supplementary Table 1**.

### Cost-Sensitive Semi-Supervised Neural Network Survival Outcome Prediction Model

Artificial neural network (ANN) (17) was used to predict patient survival outcomes (**Supplementary Figure 4**).

Considering that the characteristics of many of our lost-follow-up samples were real, but the patient’s survival status was not obtained due to various factors, we first performed pseudo-label learning (18) on the lost follow-up data to achieve data enhancement and improve the robustness and generalization ability of the model. At the same time, considering the imbalance of data categories and the credibility of pseudo-label samples, we introduced cost-sensitive learning (19) into the neural network model, and by customizing the dynamic weighted delay mean square error loss function, increasing the cost of mis-predicting dead samples and appropriately penalizing the pseudo-label samples. The dynamic weighted delay mean square error loss function is defined as follows:


(1) $$\begin{array}{r} Loss=dyn\_weight\_MSE\left(y_{1},out_{x_{1}}\right)+\alpha \left(t\right)\\ {}*dyn\_weight\_MSE\left(y_{2},out_{x_{2}}\right) \end{array}$$

Where *y*_1_ and *out*_*x*_1__ are the label and output of the neural network of the true-label samples, and *y*_2_ and are the label and output of the neural network of the pseudo-label samples.

The dynamic weighted mean square error loss function is defined as follows:


(2) $$dyn\_weight\_MSE\left(y,out_{x}\right)=Weight*MSE$$

where MSE is the batch mean square error function:


(3) $$MSE=\frac{1}{2*batchsize}\sum_{i}^{batchsize}\sum_{k}{\left(y_{k}-out_{x_{k}}\right)}^{2}$$

*Weight* is dynamically determined by the balance of each category. This was calculated once for each batch. The value of the weight is the number of positives and negatives in the real label divided by the total. The weight is defined as:


(4) Weight=(1−true_label)∗zero_weight+true_label∗one_weight    

where $\text{zero}\_\text{weight}=\frac{{\text{N}}_{\text{p}}}{\text{N}}$ , $\text{one}\_\text{weight}=\frac{{\text{N}}_{\text{n}}}{\text{N}}$ N_p_ is the total number of dead samples in each batch, N_n_ is the total number of survival samples in each batch, and N is the total number of samples in each batch.

The delay function was defined as (18):


(5) $$\alpha \left(t\right)=\{\begin{array}{cc} 0 & t<T_{1}\\ \frac{t-T_{1}}{T_{2}-T_{1}}\alpha_{f} & T_{1}\leq t\leq T_{2}\\ \alpha_{f} & t>T_{2} \end{array}$$

We designed a three-layer neural network under the framework of TensorFlow, with 117 neurons in the input layer, two hidden layers, 64 neurons in each layer, and 2 neurons in the output layer. The hyperparameters were *learning rate* = 0.002, *batchsize* = 32, *α_f_* = 3, *T*_1_ = 100, *T*_2_ = 600, and the model was built in the python3.7 environment. The specific steps were as follows:

- Train the model using labeled data, in which the ratio of surviving cases to death cases is close to 1:1 through selectively under-sampling of survival cases;
- Use the trained model to predict the label for the unlabeled data, that is, obtain the pseudo label of the unlabeled data;
- Use the label and pseudo-label data set obtained in (ii) to retrain the model and add cost-sensitive including class penalty (set weight) and pseudo-label sample penalty (set delay);
- Use the model obtained from (iii) for the final prediction of the test set.

### Lasso and Cox Survival Factor Analysis Model

The least absolute shrinkage and selection operator (LASSO) algorithm (20) is an optimization algorithm that can compress variables and reduce dimensionality and make some coefficients estimated to be zero by adding penalty constraints to the least squares estimation. We chose the best regularization coefficient by cross-validation and then input the selected features into the Cox proportional hazards regression model (21).. The model used survival outcome and survival time as dependent variables and the other variables as covariates for survival analysis, and adopted the forward step method, where the maximum number of iterations was 20, the step probability was 0.05 to enter, and 0.10 was removed, and the confidence interval for EXP(B) was 95%. The LASSO algorithm for feature selection was built in Python3.7, and the Cox model was run in SPSS 22.0.

### Logistic Regression Survival Risk Assessment Model

We used the logistic regression (LR) model (22) trained for labeled data, in which the ratio of surviving cases to death cases was close to 1:1 through selective under-sampling of survival cases, to obtain the logistic regression coefficient, namely the risk coefficient of each feature. We then used this coefficient to multiply the feature value to get the survival risk score of each patient. The model was built in the python3.7 environment. The specific steps were as follows:

1) Given the training data set: ${\left\{\left(x_{i},y_{i}\right)\right\}}_{i=1}^{n}$ , the maximum likelihood estimate (MLE) method was used to obtain the logistic regression coefficient of each feature, that was, the risk coefficient:


(6) $$l\left(w,b\right)=\sum_{i=1}^{n}ln\;p\left(y_{i}|xi;w,b\right)$$

2) The risk factor was multiplied by the eigenvalues and summed to obtain the risk score for each patient:


(7) $$z=w^{T}x=w_{0}x_{0}+w_{1}x_{1}+w_{2}x_{2}+\ldots +w_{n}x_{n}=\sum_{i=0}^{n}w_{i}x_{i}$$

Where *w*_0_, *w*_1_, …, *w_n_* are the risk coefficients of the features *x*_0_, *x*_1_, …, *x_n_*.

3) Draw a map of the patient risk score in the training set, and dig out the cut-off value of the risk score of surviving and dead patients.

4) Assess patient survival outcomes based on risk scores and thresholds:


(8) $$\left(y=1|x\right)=w_{0}x_{0}+w_{1}x_{1}+w_{2}x_{2}+\ldots +w_{n}x_{n}>T$$

(9) $$\left(y=0|x\right)=w_{0}x_{0}+w_{1}x_{1}+w_{2}x_{2}+\ldots +w_{n}x_{n}<T$$

where (*y* = 1|*x*) is the predicted outcome of death, (*y* = 0|*x*) is the predicted outcome of survival, and *T* is the survival-to-death risk score threshold.

### Other Classifiers

Decision tree (23) (DT) can be used for both classification and regression prediction of samples, the essence of it is to use a tree structure to display the entire decision-making process. Random forest (24) (RF) usually uses decision tree as the basic classifier, which is a specific implementation of Bagging algorithm, and its randomness is mainly reflected in two aspects: sample sampling and feature selection. Gradient boosting decision tree (25) (GBDT) is a decision tree generated by one iteration of gradient boosting (GB) algorithm, and it is an efficient method to solve the two-class problem. K-Nearest Neighbor (26) (KNN) is a non-explicit learning process, it uses distance metric, K value, and classification decision rules to divide the feature space, and the new samples are directly classified or forecast with the training set. Support vector machine (27) (SVM) is a common machine learning method based on statistical learning theory and risk minimization principle, its purpose is to find a hyperplane with maximized interval in vector space, which can divide samples into two categories, and has the best generalization ability.

### Statistical Analysis

SPSS software 22.0 and Python 3.7 were used for statistical analyses. Continuous data were expressed as means and standard deviations, whereas categorical data were presented as percentages. Non-normally distributed data were analyzed using the Mann-Whitney U-test. Categorical data were compared by means of the χ2 test. P values of less than 0.05 were considered to be statistically significant.

## Results

### Survival Outcome Prediction for h-SLE Patients

To predict survival outcomes, a prediction model was developed based on the characteristics of the hospitalization period and survival data during follow-up. The 1370 follow-up cases were divided into a training set (1096 cases) and a test set (274 cases) at a ratio of 8:2, grouped by survival time, and mortality rates were analyzed by counting the deaths of patients in each group (**Supplementary Table 2**). It was found that less than 20% of patients died within 15 years, and nearly 10% died within 5 years, implying that 5 years may be a watershed for survival outcomes in SLE patients.

Of the 1096 cases in the training set, there were 916 survival cases and 180 deaths, an imbalance in the data categories. To address this imbalance, we first made the survival and mortality rates close to 1:1 by selectively under sampling, and then predicted 1074 missing follow-up cases with the training model and labeled them accordingly (pseudo labels), including 467 death and 607 survival labels, indicating that dead patients accounted for a relatively high proportion of the missing follow-up data, facilitating enhanced characterization of death cases and improving the death recall rate. Finally, the pseudo-label cases and follow-up cases were sent to the cost-sensitive neural network for training.

Model inputs include not only basic patient information such as age at admission, but also clinical and serological information. The model output corresponds to the probability of patient survival and death. Surviving patients were assigned 0, and dead patients were assigned 1. Model performance was assessed by evaluating classification accuracy, sensitivity, and specificity (defined as follows):


(10) $$\text{Sensitivity}=\frac{TP}{TP+FN}$$

(11) $$\text{Specificity}=\frac{TN}{TN+FP}$$

(12) $$\text{Accuracy}=\frac{TP+TN}{TP+TN+FN+FP}$$

where TP, TN, FP, and FN denote the true positive, true negative, false positive, and false negative rates of the categories, respectively.

To highlight the effectiveness of the improved model, we compared the prediction results of several commonly used classifiers. The relevant parameters of the training cohorts and the test cohorts were shown in **Supplementary Table 3 and Supplementary Table 4**, and the receiver operating characteristic (ROC) curves of the multiple classifiers are shown in **Supplementary Figure 5**; *FP_r_* is the horizontal coordinate and *TP_r_* is the ordinate, defined as follows:


(13) $$FP_{r}=\frac{FP}{TP+TN}$$

(14) $$TP_{r}=\frac{TP}{TP+FN}$$

**Figure 1:**
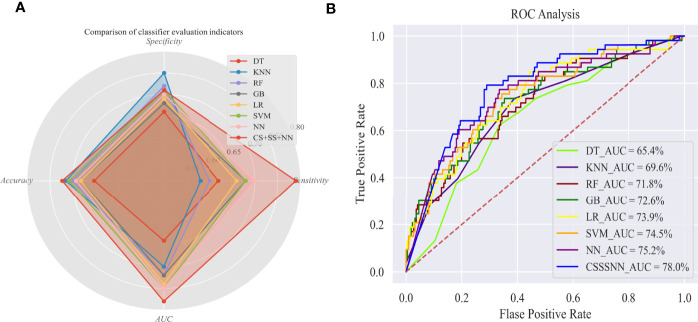
Evaluation of survival outcome prediction models in Test set. **(A)** Comparison of classifier evaluation indicators; **(B)** ROC analysis. AUC, Area under the receiver-operator characteristic curve; DT, decision tree; KNN, k-nearest neighbor; RF, random forest; GB, gradient boosting; LR, logistic regression; SVM, support vector machine; NN, neural network; CS+SS+NN, cost-sensitive semi-supervised neural network.

**Figure 1A** shows that although the k-nearest neighbor (KNN) performed well in specificity (0.738), it performed poorly in terms of sensitivity (only 0.547), which did not meet our expected goal. In terms of sensitivity, only the neural network and the improved neural network had a sensitivity close to and above 0.7, indicating that the neural network had better performance in terms of sensitivity than the other classifiers. Compared with previous neural network models, the sensitivity of our proposed model was increased by 10% through semi-supervised learning (pseudo-label learning), realizing data enhancement and cost sensitivity learning (dynamic weighted delay loss function), increase the cost of incorrectly predicting dead samples and appropriately penalize pseudo-label samples. Moreover, the specificity and accuracy of the improved model were also improved to some extent, indicating that the improved model had better robustness and generalization ability. **Figure 1B** shows that the area under the receiver operator characteristic curve (AUC) value of our proposed model was 3% higher than that of the previous neural network, and 13% higher than that of the decision tree (DT).

To identify important features in the neural network model that play crucial role in mortality, we proposed a root cause analysis method for internal verification. First, the wrong judgment rate (WJR) of the 20 prediction outcomes was analyzed, and the internal validation set was grouped according to the WJR into four subgroups: true negative group (TNG, survival judged as survival, WJR< 0.25), false positive group (FPG, survival judged as death, WJR> 0.75), true positive group (TPG, death judged as death, WJR<0.25), and false negative group (FNG, death judged as survival, WJR> 0.75). Then, we calculated the effective proportion (abnormal/involved) or mean value of each feature (Fep) in the four groups, and then defined a parameter as the death threat coefficient (Dtc):


(15) $$Dtc_{FPG-TNG}=\frac{Fep_{FPG}}{Fep_{TNG}}$$

(16) $$Dtc_{TPG-FNG}=\frac{Fep_{TPG}}{Fep_{FNG}}$$

The higher the Dtc value, the greater the threat of the feature to death. The characteristics of both coefficients simultaneously close to 1.5 and above were shown in the **Supplementary Table 5**, In terms of main description, fever and dropsy symptoms had a high Dtc value. In terms of diagnosis basis, patients with serositis had a higher risk of death. In terms of clinical manifestations on admission, the features of psychiatric symptoms, lupus headache, cylindruria, hair loss, pericarditis, and fever > 38°C, had a higher risk of death. In terms of organ involvement, Dtc values were higher in patients with neuropsychiatric and cardiopulmonary involvement. Serology tests showed higher diagnostic values for platelet (PLT), aspartate aminotransferase (AST), blood urea nitrogen (BUN), serum creatinine (Scr), and epidermal growth factor receptor (eGFR) abnormalities.

### Survival Factor Analysis for h-SLE Patients

SLE has a high degree of heterogeneity, survival status is affected by many influencing factors, and inclusion and exclusion of variables are crucial for model construction. We first tried to use PCA and PLS-DA for feature dimensionality reduction on the follow-up data set. The scatter plot of the differences between groups showed that the principal components could not be separated as can be seen by either PCA or PLSDA **(** **Supplementary Figures 6A, B** **)**. In addition, we drew a simple variable importance ranking chart according to the variable importance in the analysis results, and the results showed that the variable importance distinction was not obvious **(** **Supplementary Figures 6C, D** **)**.

Further, we developed a Cox model to identify characteristics for patient survival outcomes and survival time, In the neural network model, a relatively large number of features were input to improve the accuracy and robustness of the model; further, to analyze survival factors more effectively, we filtered the features using the least absolute shrinkage and selection operator (LASSO) algorithm before establishing the Cox model. The best regularization factor (λ= 0.0014) was selected to pass cross-validation, and the specific parameters of the 35 features selected were shown in **Supplementary Table 6**. Next, these features were input into the Cox model to analyze the survival factors of patients. The model orthogonal and consisted of 12 steps. The specific 12 variable parameters were listed in **Supplementary Table 7**.

As shown in **Supplementary Table 5**, among the 12 variables in the model, the bias regression coefficients for the seven variables of neuropathy (NP) in diagnostic basis, fever>38°C, SLEDAI at discharge, cardiopulmonary-involvement, abnormal alanine aminotransferase (ALB), abnormal blood urea nitrogen (BUN), and abnormal Anti-ds-DNA were all greater than 0, while the risk coefficients were greater than 1, with significant differences between groups (p<0.05). The partial regression coefficient of SLEDAI at discharge was 2.558, which was the only variable with a partial regression coefficient greater than 2 and a risk coefficient greater than 10, suggesting that the level of SLEDAI reflects the severity of the disease.

**Figure 2:**
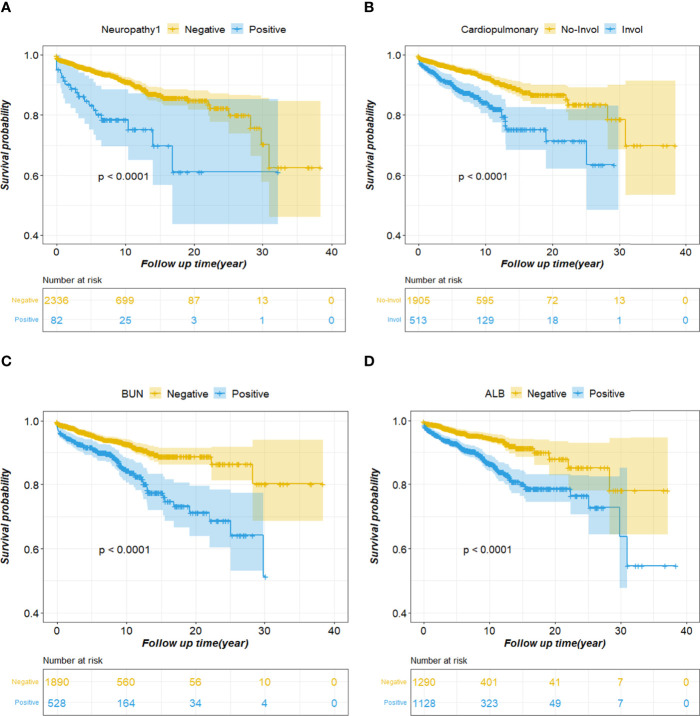
Cumulative survival curves of the survival factor. **(A)** Neuropathy; **(B)** Cardiopulmonary; **(C)** BUN; **(D)** ALB. BUN, blood urea nitrogen; ALB, albumin.

Various factors affect SLE survival, in addition to SLEDAI, cardiopulmonary and neuropsychiatric involvement, abnormal blood urea nitrogen levels, and abnormal alanine aminotransferase levels have the greatest impact on patient survival time. We performed univariate survival analysis of these four factors using the Kaplan-Meier (KM) method, and the curve depicted the characteristics of patient survival over time (**Figure 2**). Further, the probability of survival is greatly reduced in patients with neuropathy (**Figure 2A**). Moreover, cardiopulmonary impairment directly affects the quality of life and long-term prognosis of SLE patients, making it a cause of death in SLE patients (**Figure 2B**). Similarly, abnormal blood urea nitrogen (BUN) and albumin (ALB), which usually means that the patient has renal damage and liver dysfunction, respectively, affect the survival time of the patient (**Figures 2C, D**).

Machine learning-based tree models, such as random forests and gradient boosting decision trees, can show feature importance. In order to deeply analyze the factors affecting survival, the tree model was used to further analyze the survival factors (**Supplementary Figure 7**). The results of random forest model showed that ALB abnormality, admission age, PLT abnormality, and SLEDAI at discharge had a greater impact on the survival outcome of patients; the gradient boosting decision tree model results showed that admission age, ALB abnormality, and SLEDAI at discharge had a greater impact on the survival outcome of patients. Although the tree model did not consider the survival time like the Cox model in the process of building the model, the conclusions drawn were basically the same as those of the Cox model.

### Survival Risk Assessment for h-SLE Patients

However, the prediction model only predicted survival and death of patients and had difficulty distinguishing between patients at risk in the initial hospitalization period. In addition, the Cox model must include two variables, survival outcome and survival time. Taking into account the multiple factors affecting the survival outcome and the longer span of survival time, it causes great inconvenience to the patient’s risk assessment, and would also make the assessment results questionable.

**Figure 3:**
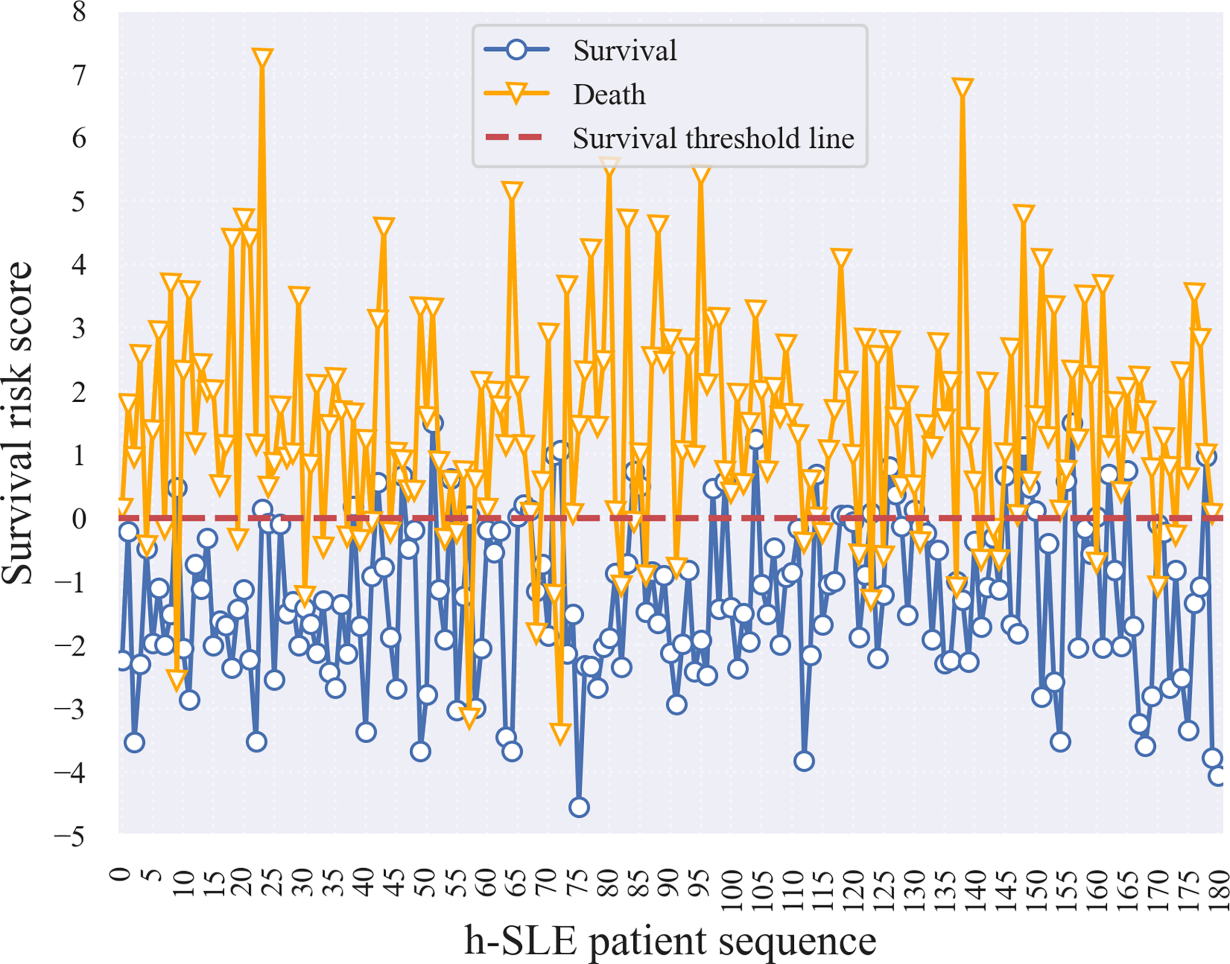
Survival risk score of h-SLE patients in Train set.

Therefore, a logistic regression (LR) model was developed to estimate the survival risk of patients in order to allow physicians to better assess the severity of the disease based on conventional measures during the initial phase of patient admission to the hospital. Risk coefficients were obtained by feeding follow-up data under selective sampling into the LR model for training (**Supplementary Table 8**). A risk coefficient greater than zero indicates that the feature has an effect on death, with larger values indicating a greater degree of effect; when the risk coefficient is less than zero, the smaller the absolute value, the lower the threat of death. For neuropathy on diagnostic basis, cranial NP, and ALB-abnormalities, risk coefficients greater than 1 indicated that these three features posed a greater threat to death. Based on the implication of the risk coefficient, the risk score reflects the severity of the patient’s illness or the risk of death; that is, the lower the patient’s risk score, the less severe the disease, the lower the risk of death, and the higher the patient risk score, the more severe the disease, the higher the risk of death. To verify this, we selected 180 surviving and dead patients from the training set, and calculated their survival risk scores (**Figure 3** **)**. Although there were some outliers in the survival risk scores obtained from the LR model for surviving and dead patients during hospitalization, there was a clear cut-off value and a clear dividing line, which we defined as the zero value of the survival threshold and a horizontal line of the zero value. The fact that the scores of surviving patients were mostly below the survival threshold, and the scores of dead patients were mostly above survival threshold, which is an important and meaningful finding that is helpful in estimating the risk of admitted patients.

**Figure 4:**
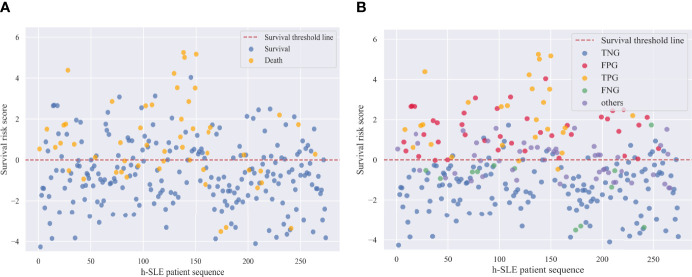
Survival risk scores of h-SLE patients in Test set. **(A)** Survival and death patients in test set; **(B)** Internal validation group of survival outcome prediction model. TNG, true negative group; FPG, false positive group; TPG, true positive group; FNG, false negative group.

In addition, survival risk scores were calculated for patients based on the characteristic risk coefficients obtained by logistic regression. **Figure 4A** shows that the cut-off line between survival and death was zero. We then divided the survival risk scores of all patients in the test set, including samples with WJR between 0.25 and 0.75, into five groups (i.e., the first four subgroups, and the fifth group “others”) (**Figure 4B**). This revealed that almost all samples in the false-negative group had survival risk scores mostly less than zero or close to zero, indicating that these patients had severe screening indicators at the beginning of their admission, but were still likely to survive under effective treatment. The majority of samples in the false-positive group had survival risk scores greater than or near zero, indicating that these patients had a higher risk of death due to unexpected complications or lack of effective treatment. Moreover, patients with WJR values between 0.25 and 0.75 had survival risk scores that fluctuated around zero, supporting these were patients that were difficult to identify by the prediction model.

### Integration and Development of Survival Analysis Model

**Figure 5:**
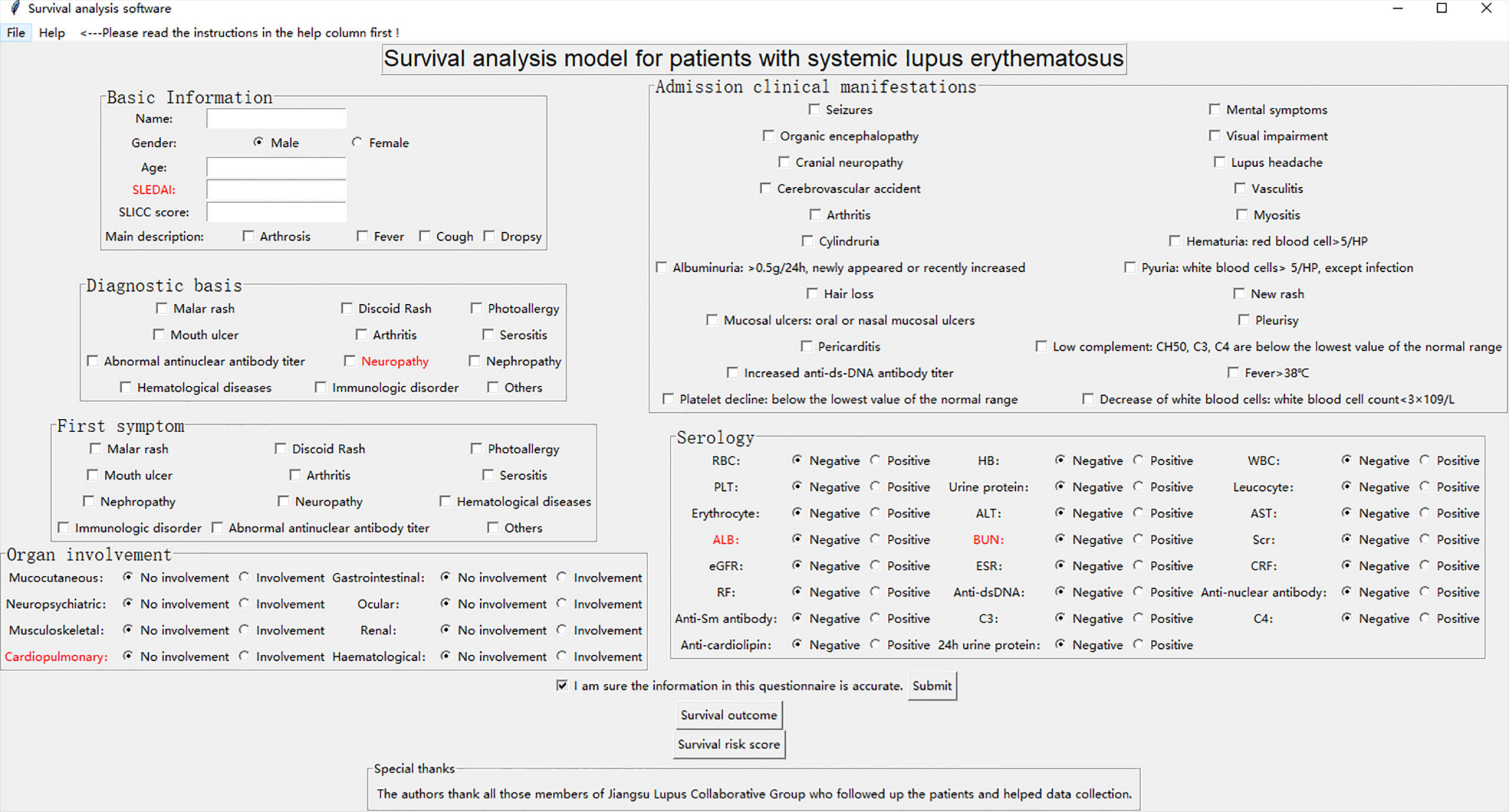
Graphical User Interface of survival analysis models.

We developed a Graphical User Interface (GUI) of survival analysis models in the python (version 3.7) environment **(** **Figure 5** **)**. The software system embedded and integrated the survival outcome prediction model based on the cost-sensitive semi-supervised neural network and the survival risk assessment model based on logistic regression, while several key indicators affecting survival time and survival outcome of h-SLE patients were red-flagged. The system automatically saves patient information (including basic information, diagnosis basis, admission clinical manifestations, first symptoms, organ involvement, serological indicators, etc.), calculates the patient’s risk score and predicts the patient’s survival outcome based on the input characteristic values (click the risk score button and survival outcome button, respectively). This not only provides a more intuitive method for survival models, but also facilitates practical clinical applications. We used a test case to further improve the demonstration of the GUI (**Supplementary Figure 8**).

## Discussion

A major difficulty in survival analysis research is the large amount of censored data, the number of patients censored patients in this paper is close to 50% (1074/2453). We analyzed whether the censored survival data might be suggestive or have some bias. Based on whether the survival outcomes were missing, we divided the data into loss-to-follow-up group and follow-up group, and conducted a difference test between the groups (**Supplementary Table 9**). The results showed that there was no significant difference between the two groups in terms of age, SLEDAI at discharge, organ involvement and serology. In terms of medication use, there was a significant difference in Prednisone and Cyclophosphamide, and we had made further statistical analysis on the medication use of patients (**Supplementary Table 10**). The results of the study showed that the lack of survival outcomes was not completely missing at random (MCAR), it depended on the situation of medication, and part of it might also be completely non-random missing (MNAR), which the patient’s family was reluctant to provide information or lost contact with the patient. In addition, a PLS-DA analysis was performed on the total data and the results indicated that the degree of distinction between the follow-up data and the censored data was not obvious **(** **Supplementary Figure 9** **)**.

Survival outcomes for lost to follow-up are categorical data rather than quantitative continuous data, and imputation and estimation methods are not ideal. We had tried to use generative adversarial network (GAN) to simulate samples to achieve data augmentation, but the results were not very satisfactory. Pseudo-label learning is a method for imputing missing values that has been shown to enable data augmentation (28). We trained the model by down-sampling to predict the survival outcome of lost samples, the accuracy and loss function of the model were shown in the **Supplementary Figure 10**.

To describe the potential advantages of the model proposed in the paper and further confirm the effectiveness of pseudo-labels, we added new evaluation indicators such as F1 score, NRI (Net Reclassification Index), IDI (Integrated Discrimination Improvement), to compare the models proposed in the study with previously published models, making the evaluation of the models more comprehensive (**Supplementary Table 11**). The results showed that our proposed model had the largest F1 score value, and the calculated NRI and IDI were greater than 0, which showed that our model achieved the best balance between precision and recall, and its prediction ability was improved compared with other models. Compared with the unmodified neural network model, the proportion of correct classification was increased by 10.8% (NRI=0.1079) and the prediction ability was improved by 8.7% (IDI=0.0874). Furthermore, we added pseudo-label data (467 death and 607 survival labels) obtained by semi-supervised learning to the down-sampled training dataset for retraining on multiple classifiers, and performed evaluation on the test set (**Supplementary Table 12**). The results showed that all classifiers had improved specificity except for KNN decreased by 1%, and except for DT decreased by 13% and SVM decreased by 2%, the sensitivity of the rest of the classifiers did not decrease, this was related to the fact that there were more surviving labels in the pseudo-labels. In addition, F1 score, NRI, and IDI also showed that the prediction ability of most classifiers had improved after adding pseudo-labels for training. In short, the results confirmed that pseudo-labels are effective, without affecting the authenticity of the data or offsetting changes in the model, which showed that the model our proposed could enhance the data and improve the prediction ability of the model.

Based on the existing data mining algorithms, the paper proposed a set of h-SLE patient survival analysis scheme, and also developed a survival analysis tool to assist doctors in assessing the patient’s survival status and self-examination of patients. SLE-related attending physicians, experts, scholars, and patients are all potential readers of this paper. On the other hand, researchers of other autoimmune and autoinflammatory diseases and artificial intelligence enthusiasts are also potential readers of this paper, because the set of survival analysis scheme proposed in the paper can be better transplanted to other complex diseases, providing new ideas and solutions for improving other diseases.

## Conclusions

Collectively, based on the survival data of h-SLE patients, the study provides technical support for clinical survival analysis of chronic diseases including SLE by using advanced data mining techniques based on characteristics and survival data of h-SLE patients. In the future, the accuracy and effectiveness of treatment should be improved by integrating clinical characteristics at the early stage of hospitalization to predict the severity of patients and prognosis.

## Data Availability Statement

The datasets used and analyzed in this study are available from the authors upon reasonable request. Requests to access the datasets should be directed to author LG - genglinyu1987@163.com.

## Ethics Statement

The studies involving human participants were reviewed and approved by The Ethics Committee of the Affiliated Drum Tower Hospital of Nanjing University Medical School. The patients/participants provided their written informed consent to participate in this study. Written informed consent was obtained from the individual(s) for the publication of any potentially identifiable images or data included in this article.

## Author Contributions

JC and WQ collected and analyzed data. LG and WQ wrote the manuscript. JLiang, WK, XX, WP, LL, MW, FD, HH, XD, HW, YZ, XQ, MWang, JW, JTao, JTan, ZD, MZ, JLi, HZ, and XF acquired clinical data. LG and LS designed the study and supervised the project. All authors made substantial intellectual contributions to conception of the work, the interpretation of data and approval of the final manuscript.

## Funding

The work was supported by National Key R&D Program of China (2020YFA0710800), the Major International (Regional) Joint Research Project (No.81720108020), National Natural Science Foundation of China (No. 81871283, 81501347, 81373198, 81471533, 81370730, and 81273304), and Nanjing Medical Science and technique Development Foundation (JQX20004).

## Conflict of Interest

The authors declare that the research was conducted in the absence of any commercial or financial relationships that could be construed as a potential conflict of interest.

## Publisher’s Note

All claims expressed in this article are solely those of the authors and do not necessarily represent those of their affiliated organizations, or those of the publisher, the editors and the reviewers. Any product that may be evaluated in this article, or claim that may be made by its manufacturer, is not guaranteed or endorsed by the publisher.
