# Supplementary material for: Development and Verify of Survival Analysis Models for Chinese Patients With Systemic Lupus Erythematosus
Source: Front Immunol. 2022 Jun 24;13:900332. doi: 10.3389/fimmu.2022.900332 (PMC9263294; doi:10.3389/fimmu.2022.900332)
Supplement: Supplementary file 1 [file DataSheet_1.pdf]

Supplementary material for

Development and verify of survival analysis models for Chinese patients with systemic lupus erythematosus

Supplementary Figures

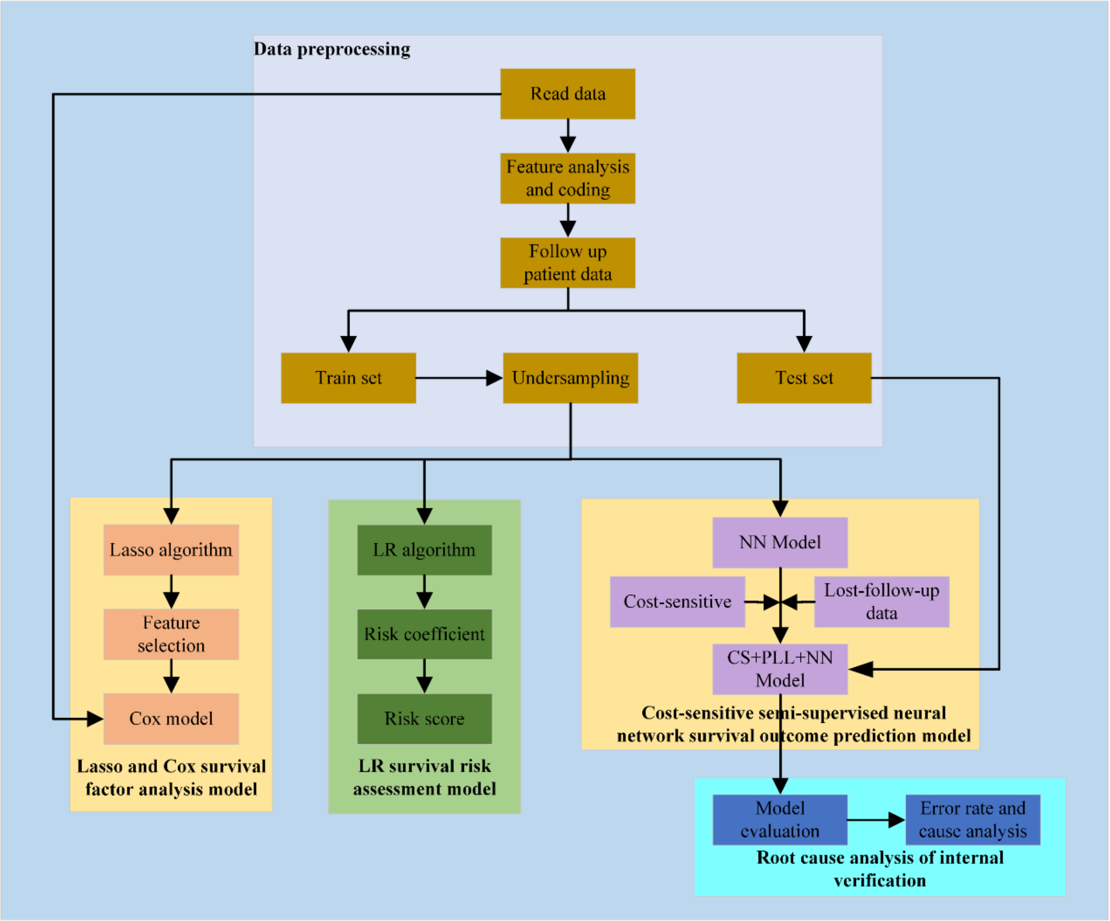

Figure 1. Technical route of survival analysis.

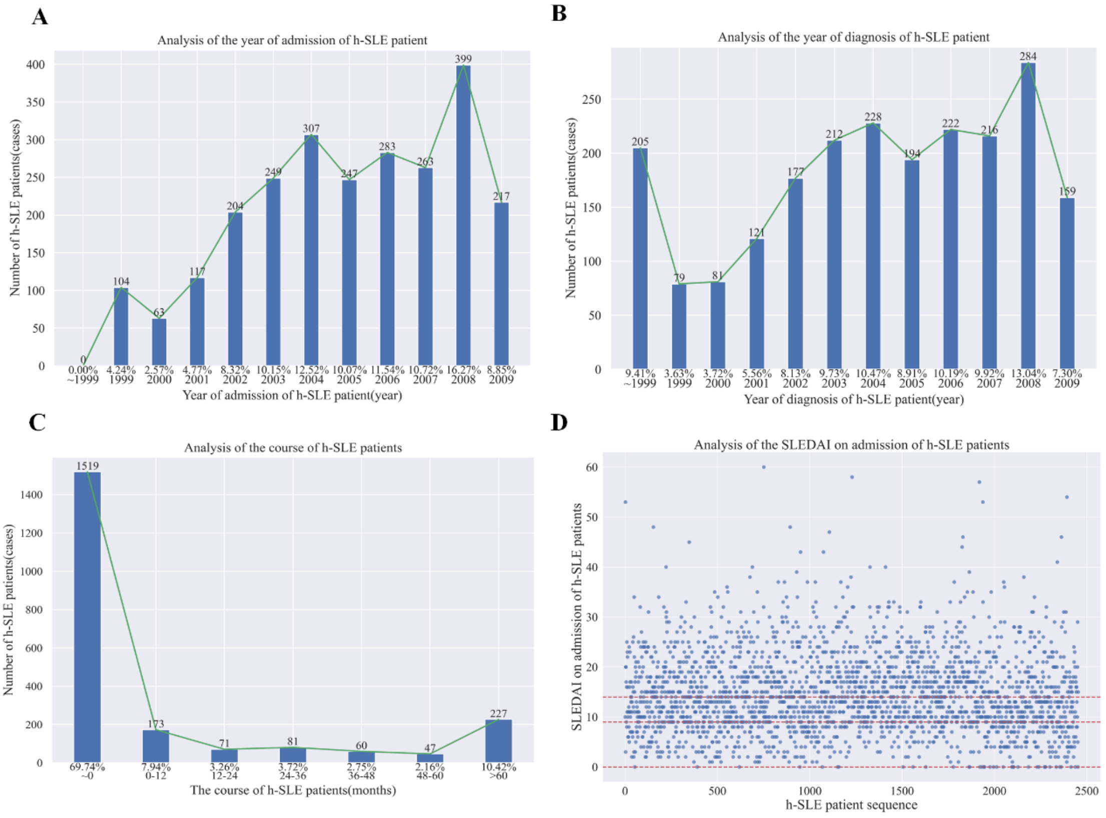

Figure 2. Basic information of h-SLE patients. (A). Year of admission. **(B).** Year of diagnosis. **(C).** Disease course. **(D).** SLEDAI on admission.

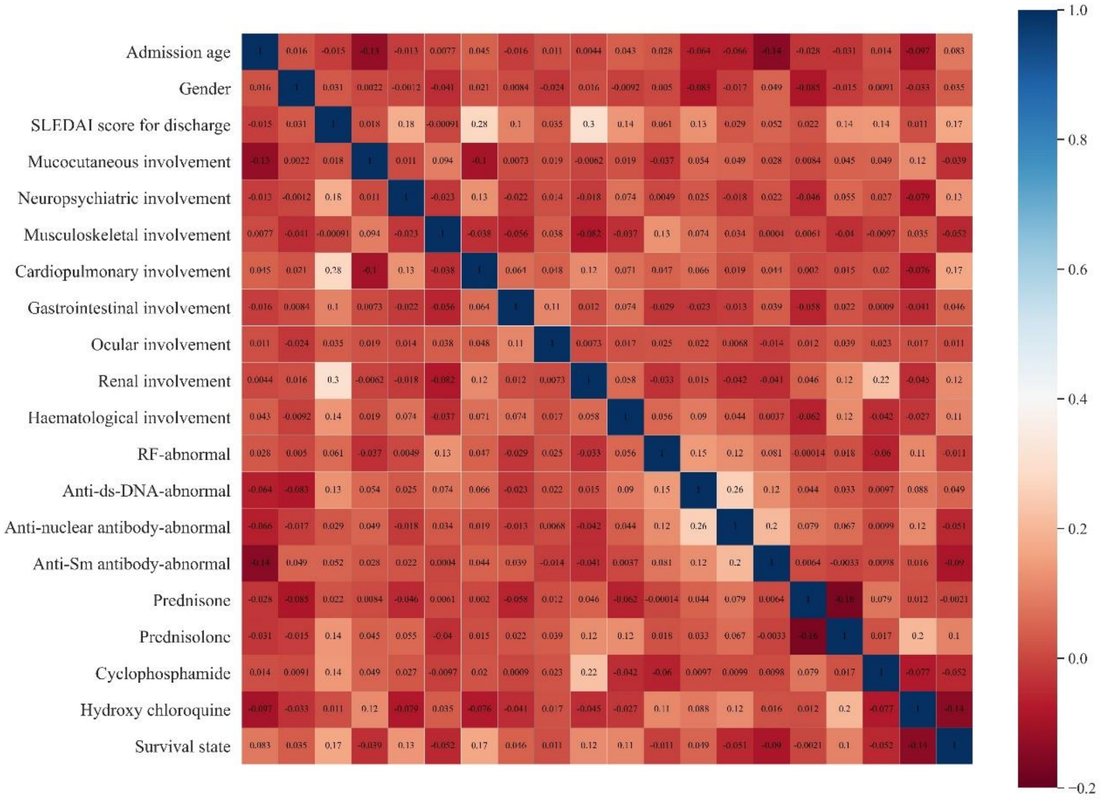

Figure 3. Correlation analysis of demographic and clinical characteristics of h-

SLE patients.

SLEDAI: SLE disease activity index; RF: rheumatoid factor.

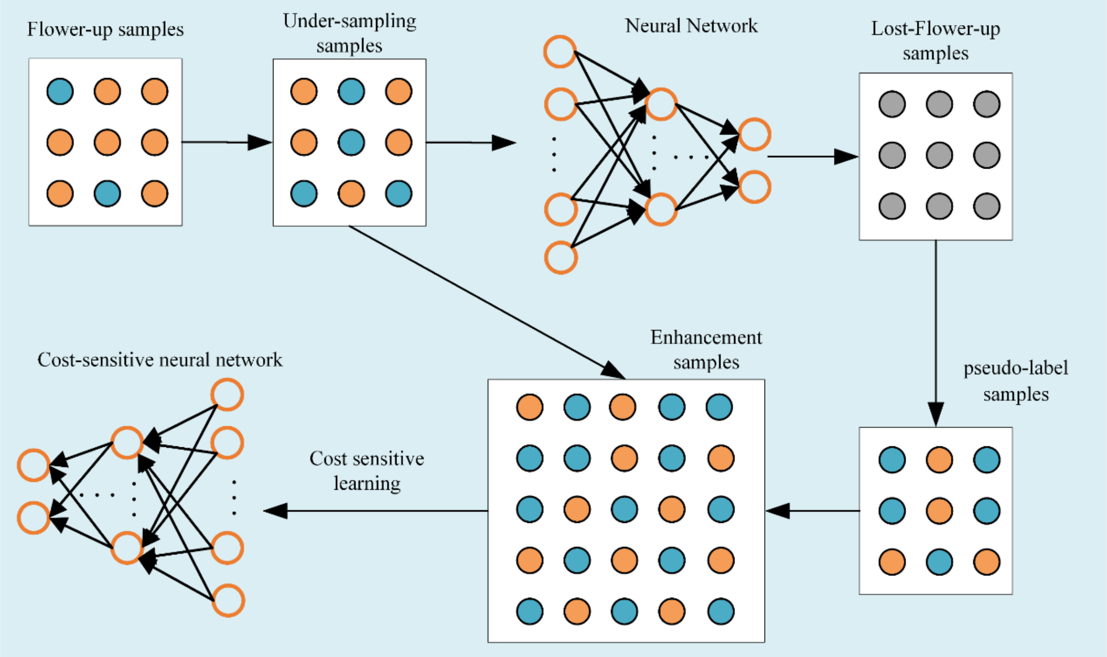

Figure 4. Survival outcome prediction model.

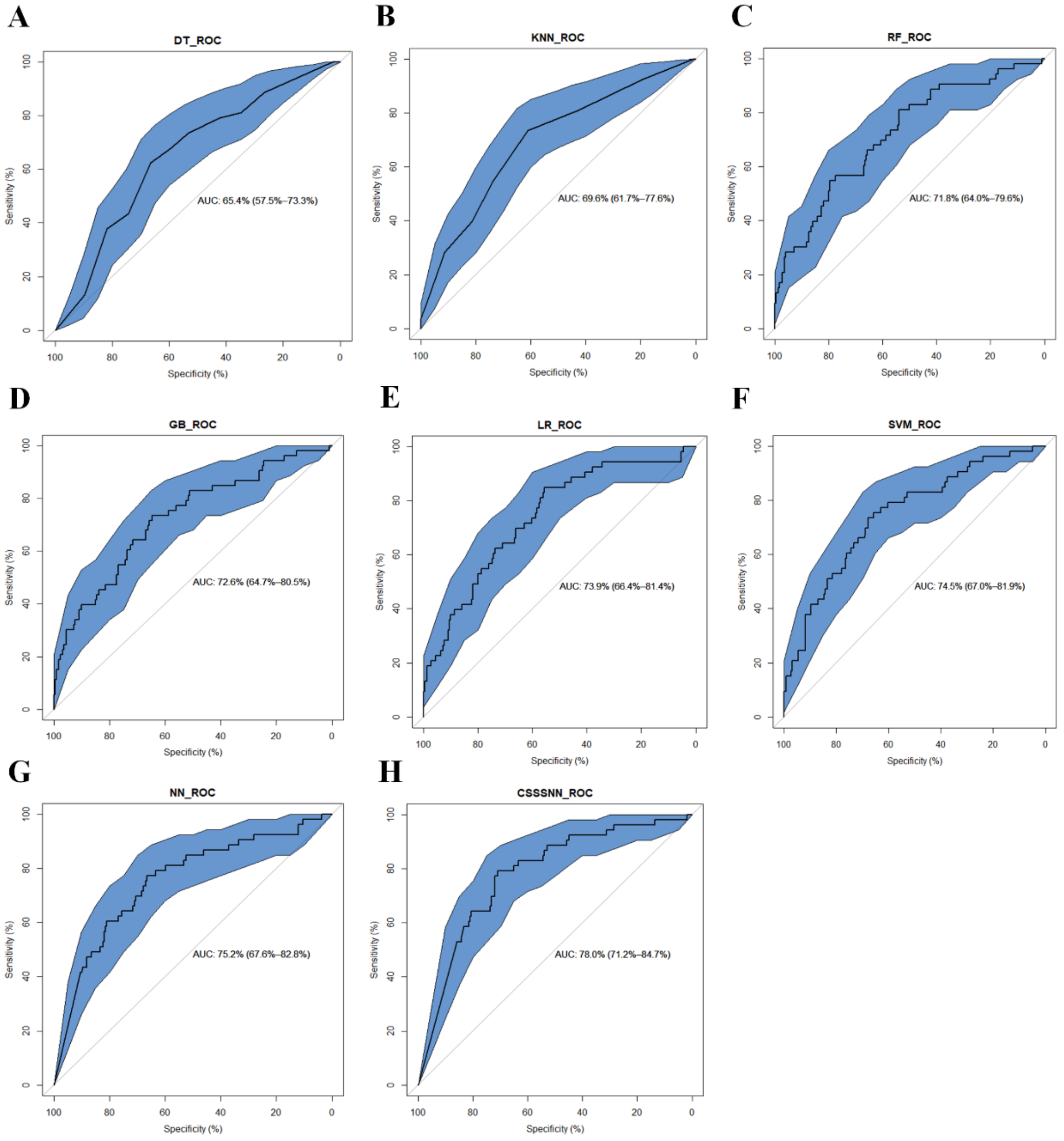

Figure 5. ROC curve analysis of the classifier. (A). DT: decision tree. **(B).** KNN: k-nearest neighbor. (**C). ** RF: random forest. **(D).** GB: gradient boosting. **(E).** LR: logistic regression. **(F).** SVM: support vector machine. **(G).** NN: neural network. **(H).** CS+SS+NN: cost-sensitive semi-supervised neural network.

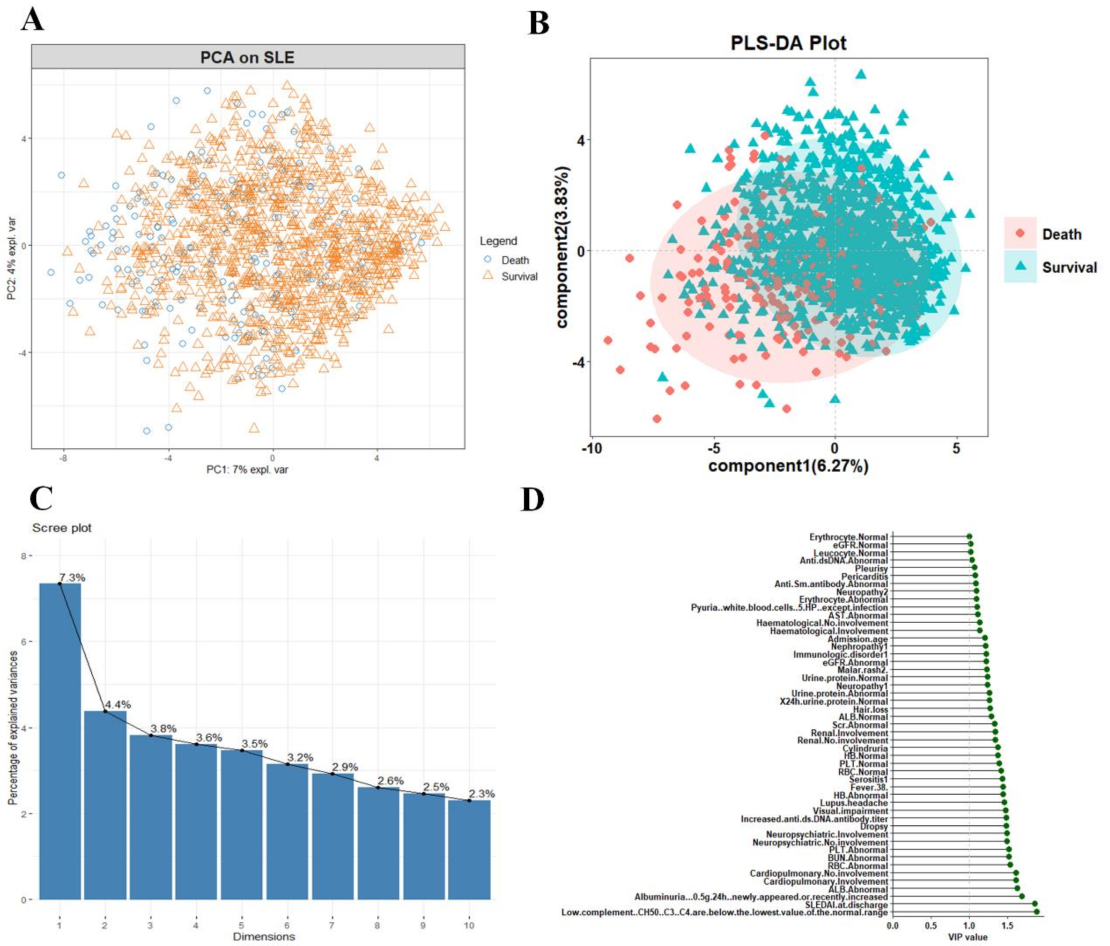

Figure 6. PCA and PLS-DA analysis of follow-up patient data. (A). PCA analysis. **(B).** PLS-DA analysis. **(C).** Principal component analysis of PCA. **(D).** Principal component analysis of PLS-DA.

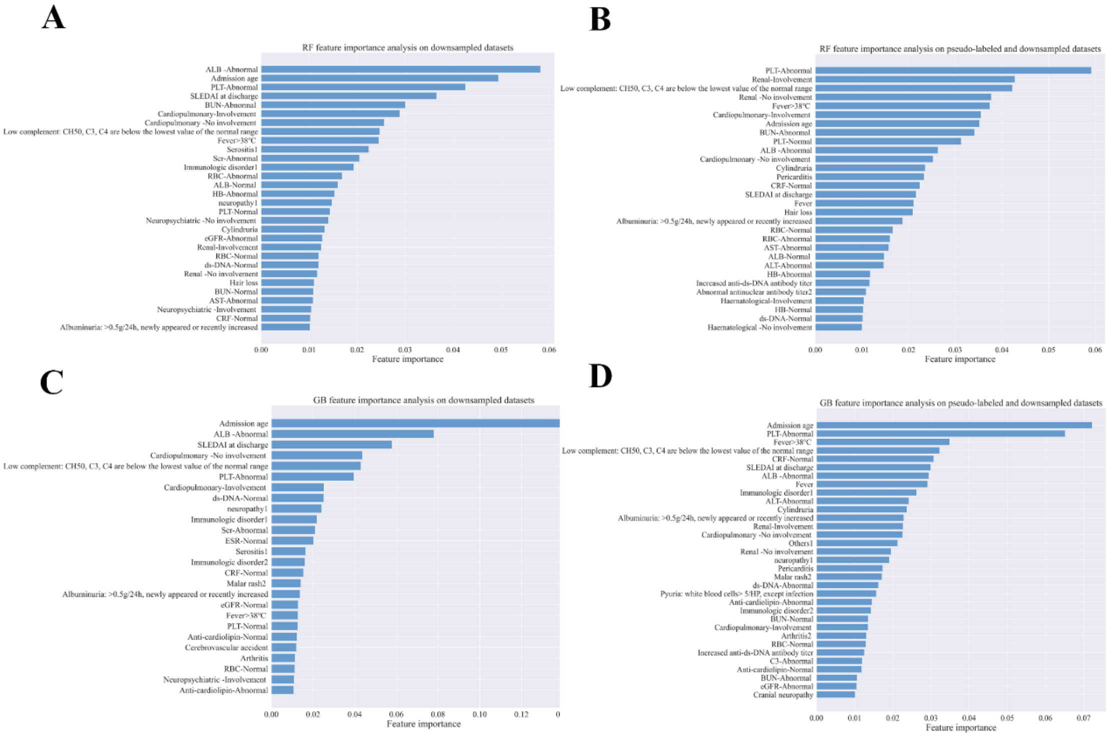

Figure 7. Feature importance (>0.01) analysis of Random forest and Gradient boosting. (A). Random Forest on down-sampling dataset. (B). Random Forest on pseudo-label and down-sampling dataset. (C). Gradient boosting on down-sampling dataset. (D). Gradient boosting on pseudo-label and down-sampling dataset.

A

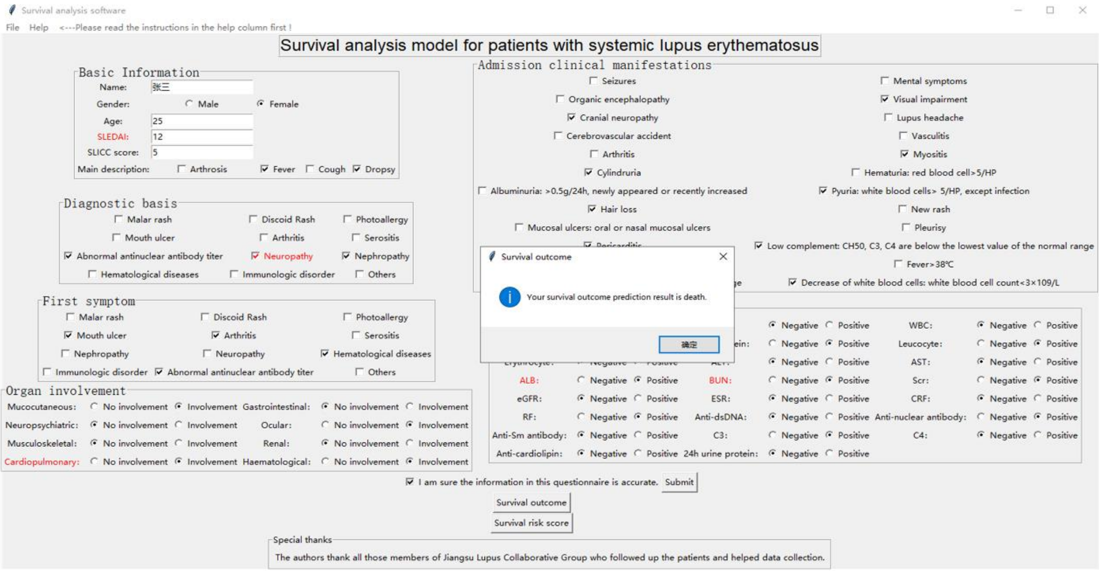

B

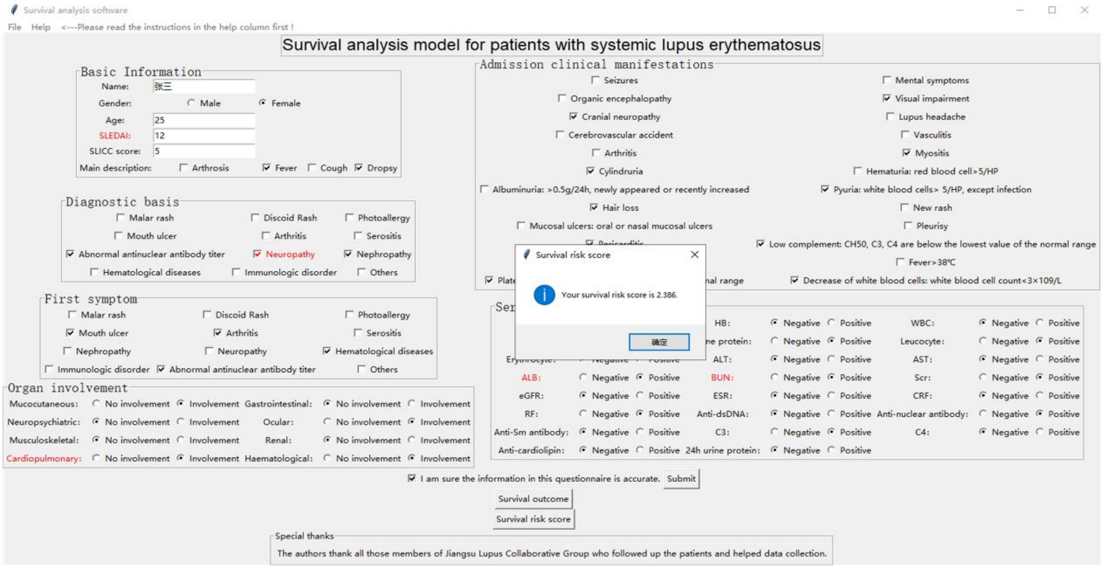

Figure 8. Case test effect of Graphical user interface. **(A).** Survival outcome prediction case. **(B).** Survival risk score case.

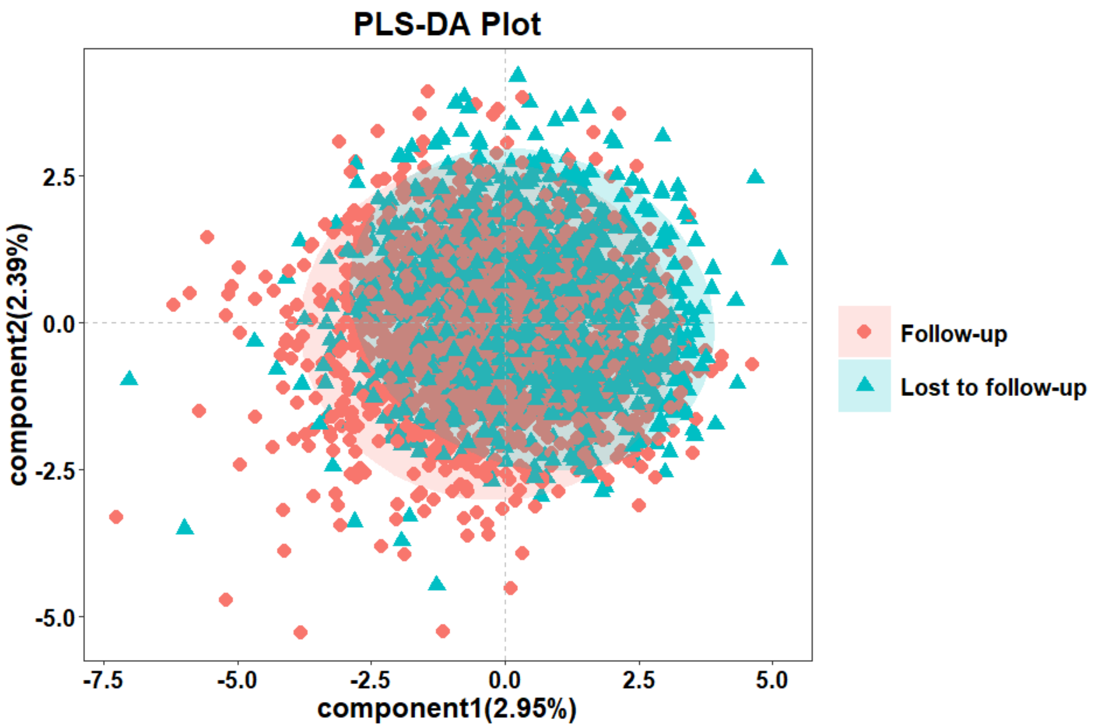

Figure 9. PLSDA analysis on total dataset

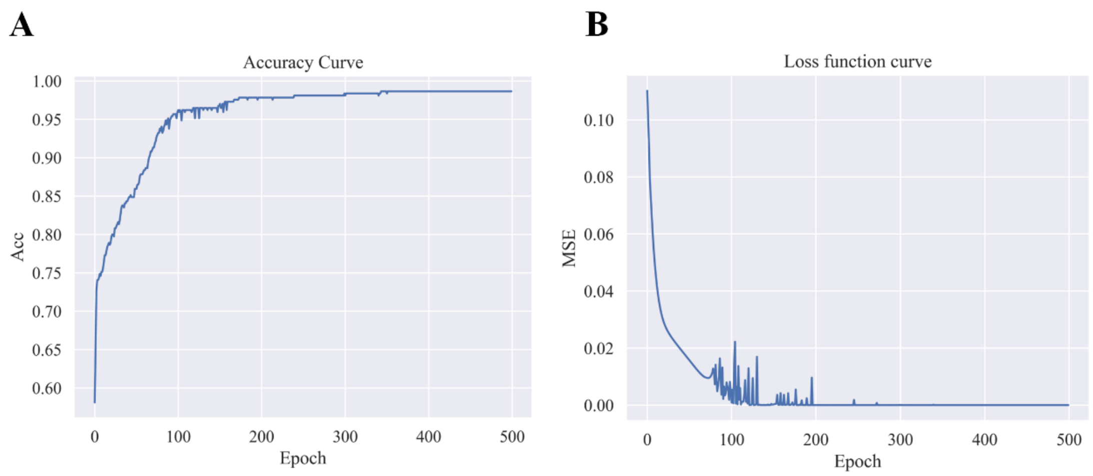

Figure 10. Accuracy and loss function curve of pseudo-label prediction model. (A). Accuracy. **(B).** Loss function.

Supplementary Tables

Table 1. Variable assignment and encoding method

Variable	Variable assignment	Encoding method
**Admission age**	Continuous type	Normalization
**Main description**	0: Arthrosis; 1: Fever; 2: Cough; 3: Dropsy	One-hot
**Diagnostic basis**	0: Malar rash[1]; 1: Discoid Rash[1]; 2: Photoallergy[1]; 3: Mouth ulcer[1]; 4: Arthritis[1]; 5: Serositis[1]; 6: Abnormal antinuclear antibody titer[1]; 7: Neuropathy[1]; 8: Nephropathy[1]; 9: Hematological diseases[1]; 10: Immunologic disorder[1]; 11: Others[1]	One-hot
**First symptom**	0: Malar rash[2]; 1: Discoid Rash[2]; 2: Photoallergy[2]; 3: Mouth ulcer[2]; 4: Arthritis[2]; 5: Serositis[2]; 6: Nephropathy[2]; 7: Neuropathy[2]; 8: Hematological diseases[2]; 9: Immunologic disorder[2]; 10: Abnormal antinuclear antibody titer[2]; 11: Others[2]	One-hot
**Admission clinical manifestations**	0: Seizures; 1: Mental symptoms; 2: Organic encephalopathy; 3: Visual impairment; 4: Cranial neuropathy; 5: Lupus headache; 6: Cerebrovascular accident; 7: Vasculitis; 8: Arthritis; 9: Myositis; 10: Cylindruria; 11: Hematuria: red blood cell>5/HP; 12: Albuminuria: >0.5g/24h, newly appeared or recently increased; 13: Pyuria: white blood cells> 5/HP, except infection; 14: Hair loss; 15: New rash; 16: Mucosal ulcers: oral or nasal mucosal ulcers; 17: Pleurisy; 18: Pericarditis; 19: Low complement: CH50, C3, C4 are below the lowest value of the normal range; 20: Increased anti-ds-DNA antibody titer; 21: Fever>38℃； 22: Platelet decline: below the lowest value of the normal range; 23: Decrease of white blood cells: white blood cell count<3×109/L	One-hot
**SLEDAI at discharge**	Continuous type	Normalization
**Mucocutaneous**	0：No involvement；1：Involvement	One-hot
**Neuropsychiatric**	0：No involvement；1：Involvement	One-hot
**Musculoskeletal**	0：No involvement；1：Involvement	One-hot
**Cardiopulmonary**	0：No involvement；1：Involvement	One-hot
**Gastrointestinal**	0：No involvement；1：Involvement	One-hot
**Ocular**	0：No involvement；1：Involvement	One-hot
**Renal**	0：No involvement；1：Involvement	One-hot
**Haematological**	0：No involvement；1：Involvement	One-hot
**SLICC score**	Continuous type	Normalization
**RBC**	0：Normal；1：Abnormal	One-hot
**HB**	0：Normal；1：Abnormal	One-hot
**WBC**	0：Normal；1：Abnormal	One-hot
**PLT**	0：Normal；1：Abnormal	One-hot
**Urine protein**	0：Normal；1：Abnormal	One-hot
**Leucocyte**	0：Normal；1：Abnormal	One-hot

**Erythrocyte**	0：Normal；1：Abnormal	One-hot
**ALT**	0：Normal；1：Abnormal	One-hot
**AST**	0：Normal；1：Abnormal	One-hot
**ALB**	0：Normal；1：Abnormal	One-hot
**BUN**	0：Normal；1：Abnormal	One-hot
**Scr**	0：Normal；1：Abnormal	One-hot
**eGFR**	0：Normal；1：Abnormal	One-hot
**ESR**	0：Normal；1：Abnormal	One-hot
**CRF**	0：Normal；1：Abnormal	One-hot
**RF**	0：Normal；1：Abnormal	One-hot
**Anti-dsDNA**	0：Normal；1：Abnormal	One-hot
**Anti-nuclear antibody**	0：Normal；1：Abnormal	One-hot
**Anti-Sm antibody**	0：Normal；1：Abnormal	One-hot
**C3**	0：Normal；1：Abnormal	One-hot
**C4**	0：Normal；1：Abnormal	One-hot
**Anti-cardiolipin**	0：Normal；1：Abnormal	One-hot
**24h urine protein**	0：Normal；1：Abnormal	One-hot

SLEDAI: SLE disease activity index; SLICC: Systemic lupus international collaborating clinics; RBC: Red blood cell; HB: Hemoglobin; WBC: White blood cell; PLT: Platelets; ALT: Alanine aminotransferase; AST: Aspartate aminotransferase; ALB: Albumin; BUN: Blood urea nitrogen; Scr: Serum creatinine; eGFR: Epidermal growth factor receptor; ESR: Erythrocyte sedimentation rate; CRF: Chronic renal failure; RF: Rheumatoid factor; C3: Serum complement C3; C4: Serum complement C4.

Table 2. Mortality analysis of h-SLE patients

Survival time	Mortality in Training set	Mortality in Test set	Overall mortality
**0-5 years**	92/1096=**8.4%**	28/274=**10.2%**	120/1370=**8.76%**
**5-10 years**	61/1096=**5.6%**	20/274=**7.3%**	81/1370=**5.9%**
**10-15 years**	27/1096=**2.5%**	5/274=**1.8%**	32/1370=**2.3%**
**0-15 years**	180/1096=**16%**	53/274=**19%**	253/1370=**18%**

Table 3. Performance evaluation of multi-classifier on training cohort

Classifier	Sensitivity	Specificity	Accuracy	F1-score
**DT**	0.8444	0.7895	0.8162	0.8162
**KNN**	0.6278	0.8053	0.7189	0.7164
**RF**	0.8389	0.9474	0.8946	0.8942
**GB**	0.9889	1.0000	0.9946	0.9946
**LR**	0.8056	0.8421	0.8243	0.8242
**SVM**	0.8944	0.9263	0.9108	0.9108
**NN**	0.9500	0.9895	0.9703	0.9702
**CS+SS+NN**	0.9877	0.9962	0.9924	0.9924

DT: decision tree; KNN: k-nearest neighbor; RF: random forest; GB: gradient boosting; LR: logistic regression; SVM: support vector machine; NN: neural network; CS+SS+NN: cost-sensitive semi-supervised neural network.

Table 4. Performance evaluation of multi-classifier on test cohort

Classifier	Sensitivity	Specificity	Accuracy	AUROC (95% CI)
**DT**	0.679	0.593	0.610	0.654(0.575-0.733)
**KNN**	0.547	0.738	0.701	0.696(0.617-0.776)
**RF**	0.642	0.661	0.657	0.718(0.640-0.796)
**GB**	0.642	0.670	0.664	0.726(0.647-0.805)
**LR**	0.642	0.688	0.679	0.739(0.664-0.814)
**SVM**	0.660	0.701	0.693	0.745(0.670-0.819)
**NN**	0.698	0.692	0.693	0.752(0.676-0.828)
**CS+SS+NN**	0.792	0.706	0.723	0.780(0.712-0.847)

AUROC: Area under the receiver-operator characteristic curve; DT: decision tree; KNN: k-nearest neighbor; RF: random forest; GB: gradient boosting; LR: logistic regression; SVM: support vector machine; NN: neural network; CS+SS+NN: cost-sensitive semi-supervised neural network.

Table 5. Root cause analysis of internal validation group

Variable	TNG	FPG	TPG	FNG	$Dtc_{FPG-TNG}$	$Dtc_{TPG-FNG}$
**Fever**	0.214	0.450	0.481	0.167	2.105	2.889
**Dropsy**	0.083	0.225	0.259	0.083	2.719	3.111
**Serositis[2]**	0.014	0.050	0.111	0.083	3.625	1.333
**Mental symptoms**	0.234	0.375	0.407	0.167	1.559	2.444
**Lupus headache**	0.014	0.125	0.259	0.083	9.063	3.111
**Cylindruria**	0.076	0.225	0.407	0.083	2.966	4.889
**Hair loss**	0.221	0.550	0.778	0.333	2.492	2.333
**Pleurisy**	0.172	0.450	0.444	0.333	2.610	1.333
**Fever>38°C**	0.179	0.400	0.593	0.083	2.231	7.111
**Neuropsychiatric[I]**	0.034	0.100	0.222	0.000	2.900	endless
**Cardiopulmonary[I]**	0.117	0.400	0.481	0.25	3.411	1.926
**PLT- Abnormal**	0.207	0.500	0.556	0.333	2.417	1.667
**AST- Abnormal**	0.138	0.300	0.481	0.25	2.175	1.926
**BUN- Abnormal**	0.152	0.475	0.481	0.167	3.131	2.889
**Scr- Abnormal**	0.103	0.250	0.407	0.167	2.417	2.444
**eGFR- Abnormal**	0.082	0.225	0.370	0.083	2.719	4.444

Dtc: Death threat coefficient; [2]First symptom; [I] Involvement; TNG: true negative group; FPG: false positive group; TPG: true positive group; FNG: false negative group; PLT: platelets; AST: aspartate aminotransferase; BUN: blood urea nitrogen; Scr: serum creatinine; eGFR: epidermal growth factor receptor.

Table 6. Feature parameters filtered by Lasso

Variable	Survival	Death	P-value	Score	Coefficient
**Admission age**	34.21±12.12	37.07±13.92	0.084169	2.983	0.020189
**Fever**	350(30.78)	83(35.62)	0.330290	0.948	0.030548
**Serositis[1]**	155(13.63)	66(28.33)	4.1836E-9	34.536	0.081609
**Nephropath[1]**	31(2.73)	20(8.58)	0.000002	22.649	0.245363
**Immunologic disorder[1]**	773(67.99)	132(56.65)	0.132212	2.266	-0.08878
**Malar rash[2]**	414(36.41)	88(37.77)	0.959479	0.003	0.031657
**Immunologic disorder[2]**	65(5.72)	6(2.58)	0.236074	1.404	-0.00702
**Abnormal antinuclear antibody titer[2]**	75(6.60)	7(3.00)	0.146037	2.113	-0.08629
**Others[2]**	42(3.69)	4(1.72)	0.350993	0.870	-0.11808
**Mental symptoms**	349(30.69)	87(37.34)	0.098352	2.732	0.027918

**Cranial neuropathy**	19(1.67)	9(3.86)	0.044251	4.047	0.09702
**Arthritis**	322(28.32)	62(26.61)	0.733999	0.115	0.006279
**Cylindruria**	137(12.05)	57(24.46)	0.000003	22.020	0.062297
**Albuminuria: >0.5g/24h, newly appeared or recently increased**	294(25.86)	41(17.60)	0.032300	4.582	-0.02482
**Hematuria: red blood cell>5/HP**	117(10.29)	16(6.78)	0.456611	0.554	-0.04443
**Pleurisy**	132(11.61)	32(13.73)	0.092610	2.828	-0.03863
**Low complement: CH50, C3, C4 are below the lowest value of the normal range**	402(35.36)	46(19.74)	0.000574	11.858	-0.07554
**Fever>38℃**	298(26.21)	102(43.78)	0.000003	21.880	0.06777
**SLEDAI at discharge**	5.69±6.08	8.77±10.04	2.2773E-9	35.721	0.033351
**Cardiopulmonary-Involvement**	199(17.50)	83(35.62)	1.5734E-9	36.441	0.109779
**Cardiopulmonary- No involvement**	938(82.50)	150(64.38)	1.5734E-9	36.441	-1.3E-17
**RBC- Normal**	614(54.00)	86(36.91)	0.000106	15.024	-0.02949
**PLT- Abnormal**	307(27.00)	108(46.35)	0.000005	20.950	0.092663
**Leucocyte - Normal**	669(58.84)	150(64.38)	0.897803	0.016	0.035427
**ALT- Abnormal**	173(15.22)	56(24.03)	0.006761	7.336	0.045252
**ALB- Abnormal**	491(43.18)	152(65.24)	2.0224E-9	35.952	0.113658
**BUN- Abnormal**	216(19.00)	85(36.48)	9.4976E-9	32.941	0.049461
**Scr- Abnormal**	157(13.81)	64(27.47)	7.0945E-8	29.039	0.121756
**eGFR- Abnormal**	139(12.23)	56(24.03)	9.9124E-7	23.945	0.012219
**ESR- Normal**	196(17.24)	20(8.58)	0.000257	13.363	-0.01548
**CRF- Normal**	486(42.74)	74(31.76)	0.000663	11.590	-0.03918
**CRF- Abnormal**	281(24.71)	75(32.19)	0.004874	7.926	0.000536
**ds-DNA-Normal**	359(31.57)	66(28.33)	0.134088	2.245	-0.0391
**ds-DNA-Abnormal**	589(51.80)	135(57.94)	0.006717	7.347	0.010822
**Anti-cardiolipin - Normal**	293(25.77)	51(21.89)	0.560203	0.339	-0.01719

Data are mean ± SD, n/N (%), Data are mean ± SD, n (%), where n is the total number of patients with valid data in each group. P-values are calculated by chi-square test. [1]Diagnostic basis; [2]First symptom; SLEDAI: SLE disease activity index; SLICC: Systemic lupus international collaborating clinics; RBC: Red blood cell; PLT: Platelets; ALT: Alanine aminotransferase; ALB: Albumin; BUN: Blood urea nitrogen; Scr: Serum creatinine; eGFR: Epidermal growth factor receptor; ESR: Erythrocyte sedimentation rate; CRF: Chronic renal failure.

Table 7. Variables in the COX model

Variable	B	SE	P-value	EXP(B) (confidence interval)
**Neuropathy[1]**	0.927	0.246	0.00016	2.528 (1.562, 4.092)
**Immunologic disorder[1]**	-0.553	0.163	0.00070	0.575 (0.418, 0.792)
**Albuminuria: >0.5g/24h, newly appeared or recently increased**	-0.507	0.232	0.02928	0.602 (0.382, 0.950)
**Low complement: CH50, C3, C4 are below the lowest value of the normal range**	-0.686	0.214	0.00135	0.054 (0.331, 0.766)

**Fever>38°C**	0.373	0.145	0.01028	1.453 (1.092, 1.932)
**SLEDAI at discharge**	2.558	0.543	0.00000	12.904 (4.453, 37.390)
**Cardiopulmonary- Involvement**	0.529	0.154	0.00059	1.697 (1.255, 2.295)
**ALB- Abnormal**	0.466	0.160	0.00364	1.593 (1.164, 2.180)
**BUN- Abnormal**	0.486	0.149	0.00114	1.625 (1.213, 2.177)
**ESR- Normal**	-0.767	0.259	0.00299	0.464 (0.280, 0.770)
**CRF-Normal**	-0.432	0.156	0.00562	0.649 (0.478, 0.881)
**Anti-dsDNA-Abnormal**	0.421	0.167	0.01139	1.524 (1.100, 2.112)

B: partial regression coefficient; EXP(B): relative risk coefficient; SE: standard error; [1]Diagnostic basis; Statistical analysis was performed with the χ2 test; The confidence level was 0.95. SLEDAI: SLE disease activity index; ALB: albumin; BUN: blood urea nitrogen; ESR: erythrocyte sedimentation rate; CRF: chronic renal failure.

Table 8. Risk coefficient of characteristic

**Variable**	**risk coefficient**
Admission age	0.604707
Arthrosis	0.224081
Fever	0.598162
Cough	-0.58383
Dropsy	0.659627
Malar rash[1]	-0.14587
Discoid Rash[1]	-0.08939
Photoallergy[1]	-0.26668
Mouth ulcer[1]	0.282705
Arthritis[1]	-0.11446
Serositis[1]	0.53914
Abnormal antinuclear antibody titer[1]	-0.04328
neuropathy[1]	**1.234376**
nephropathy[1]	-0.07246
Hematological diseases[1]	-0.12932
Immunologic disorder[1]	-0.78422
Others[1]	-0.5229
Malar rash[2]	0.978433
Discoid Rash[2]	-0.02237
Photoallergy[2]	0.23211
Mouth ulcer[2]	0.107663
Arthritis[2]	-0.1002
Serositis[2]	0.263955
Nephropath[2]	0.135992
Neuropathy[2]	0.629056
Hematological diseases[2]	-0.54046
Immunologic disorder[2]	-0.02037
Abnormal antinuclear antibody titer[2]	-0.74609
Others[2]	-0.53582

Seizures	0.43236
Mental symptoms	0.523063
Organic encephalopathy	-0.50246
Visual impairment	0.416273
Cranial neuropathy	**1.085752**
Lupus headache	-0.49565
Cerebrovascular accident	0.575124
Vasculitis	0.408975
Arthritis	0.705748
Myositis	0.201655
Cylindruria	0.649167
Hematuria: red blood cell>5/HP	-0.34456
Albuminuria: >0.5g/24h, newly appeared or recently increased	-0.80926
Pyuria: white blood cells> 5/HP, except infection	-0.97888
Hair loss	-0.19051
New rash	0.074821
Mucosal ulcers: oral or nasal mucosal ulcers	0.075181
Pleurisy	-0.67569
Pericarditis	0.07842
Low complement: CH50, C3, C4 are below the lowest value of the normal range	-0.25229
Increased anti-ds-DNA antibody titer	-0.51894
Fever>38℃	0.697167
Platelet decline: below the lowest value of the normal range	0.482126
Decrease of white blood cells: white blood cell count<3×109/L	-0.63321
SLEDAI at discharge	0.624373
Mucocutaneous- Involvement	0.162092
Mucocutaneous-No involvement	-0.22066
Neuropsychiatric -Involvement	0.094218
Neuropsychiatric -No involvement	-0.15278
Musculoskeletal-Involvement	-0.11138
Musculoskeletal -No involvement	0.052813
Cardiopulmonary-Involvement	0.404468
Cardiopulmonary -No involvement	-0.46303
Gastrointestinal-Involvement	0.161393
Gastrointestinal -No involvement	-0.21996
Ocular-Involvement	0.060985
Ocular -No involvement	-0.11955
Renal-Involvement	0.221796
Renal -No involvement	-0.28036
Haematological-Involvement	-0.10924
Haematological -No involvement	0.05068
SLICC score	0.062946
RBC-Normal	-0.36371
RBC-Abnormal	-0.45846
HB-Normal	-0.39603
HB-Abnormal	0.050891
WBC-Normal	0.223414
WBC-Abnormal	0.369566
PLT-Normal	-0.39817
PLT-Abnormal	0.229056
Urine protein-Normal	0.483437
Urine protein-Abnormal	0.26017

Leucocyte-Normal	0.551281
Leucocyte-Abnormal	0.412458
Erythrocyte-Normal	0.108884
Erythrocyte-Abnormal	0.264311
ALT-Normal	0.085716
ALT-Abnormal	0.552766
AST-Normal	-0.19432
AST-Abnormal	-0.0579
ALB-Normal	0.637841
ALB -Abnormal	**1.070925**
BUN-Normal	-0.18966
BUN-Abnormal	0.315402
Scr-Normal	-0.29292
Scr-Abnormal	0.835315
eGFR-Normal	0.869232
eGFR-Abnormal	0.687288
ESR-Normal	-0.79423
ESR-Abnormal	-0.62611
CRF-Normal	-0.33373
CRF-Abnormal	0.365666
RF-Normal	0.110966
RF-Abnormal	-0.09288
ds-DNA-Normal	-0.24424
ds-DNA-Abnormal	0.910696
Anti-nuclear antibody-Normal	-0.2348
Anti-nuclear antibody-Abnormal	-0.23331
Anti-Sm antibody-Normal	-0.0342
Anti-Sm antibody-Abnormal	-0.08607
C3-Normal	0.00617
C3-Abnormal	-0.3168
C4-Normal	-0.35013
C4-Abnormal	-0.01609
Anti-cardiolipin-Normal	-0.6677
Anti-cardiolipin-Abnormal	-0.51955
24h urine protein-Normal	-0.17962
24h urine protein-Abnormal	-0.18439

[1]Diagnostic basis; [2]First symptom; SLEDAI: SLE disease activity index; SLICC: Systemic lupus international collaborating clinics; RBC: Red blood cell; HB: Hemoglobin; WBC: White blood cell; PLT: Platelets; ALT: Alanine aminotransferase; AST: Aspartate aminotransferase; ALB: Albumin; BUN: Blood urea nitrogen; Scr: Serum creatinine; eGFR: Epidermal growth factor receptor; ESR: Erythrocyte sedimentation rate; CRF: Chronic renal failure; RF: Rheumatoid factor; C3: Serum complement C3; C4: Serum complement C4.

Table 9. Demographic and clinical characteristics of total h-SLE patients

**Variable**	**Total (N=2444)**	**Follow-up (N=1370)**	**Lost-follow-up (N=1074)**	**P value**
**Mean Age ± SD (years)**	34.86±12.42	34.70±12.49	35.06±12.32	0.4487
**Female (%)-n (%)**	2263(92.59)	1265(92.34)	998(92.92)	0.6362

**Male (%)-n (%)**	181(7.41)	105(7.66)	76(7.08)	0.6362
**SLEDAI on admission**	14.61±7.97	14.55±8.25	14.68±7.60	0.3589
**SLEDAI at discharge**	6.18±6.58	6.21±7.01	6.12±5.99	0.1768
**Organ involvements-n (%)**				
**Mucocutaneous**	1589(65.02)	913(66.64)	676(62.94)	0.0628
**Neuropsychiatric**	150(6.14)	92(6.72)	58(5.40)	0.2079
**Musculoskeletal**	1334(54.58)	743(54.23)	588(54.75)	0.8315
**Cardiopulmonary**	521(21.32)	282(20.58)	239(22.25)	0.3419
**Gastrointestinal**	118(4.83)	69(5.04)	49(4.56)	0.6544
**Haematological**	1150(47.05)	616(44.96)	534(49.72)	0.0216
**Serology-n (%)**				
**Anti-dsDNA positive**	1316(53.85)	724(52.85)	592(55.12)	0.2808
**Anti-Sm positive**	729(29.83)	411(30.00)	318(29.61)	0.8688
**Anti-cardiolipin positive**	265(10.84)	161(11.75)	104(9.68)	0.1172
**RF positive**	544(22.26)	288(21.02)	256(23.84)	0.1072
**Medications-n (%)**				
**Prednisone**	1830(74.88)	972(70.95)	858(79.89)	**0.0000**
**Prednisolone**	1058(43.29)	590(43.07)	468(43.58)	0.8327
**Cyclophosphamide**	955(39.08)	573(41.82)	382(35.57)	**0.0019**
**Hydroxy chloroquine**	810(33.14)	475(34.67)	335(31.19)	0.0766

SLEDAI: SLE disease activity index; RF: rheumatoid factor. Data are presented as mean ± SD, n (%), where n is the total number of patients with valid data in each group. Statistical analysis was performed using the Mann-Whitney U-test and the $\chi 2$ test.

Table 10. Statistical analysis on the medication use of patients

Medications	follow-up	Lost-follow-up	P value	survival	Death	P value
**Prednisone**	972	858	**0.0000**	806	166	0.9761
**Prednisolone**	590	468	0.8327	463	127	**0.0001**
**Cyclophosphamide**	573	382	**0.0019**	489	84	0.0590
**Hydroxy chloroquine**	475	335	0.0766	427	48	**0.0000**
**Dexamethasone**	236	93	**0.0000**	201	35	0.3772
**Chloroquine**	89	111	**0.0008**	78	11	0.2887
**Methotrexate**	58	42	0.7663	50	8	0.6261
**Tripterygium**	67	60	0.4980	54	13	0.7125
**Mycophenolate**	44	42	0.4121	33	11	0.2185

Table 11. Further performance evaluation of model performance on down-sampling dataset

Classifier	F1-score	NRI	IDI
**DT**	0.6505	0.2263[0.0636-0.389]<br>p-value: 0.0064	0.3043[0.1644-0.4442]<br>p-value: 2e-05
**KNN**	0.7246	0.2136[0.0623-0.3649]<br>p-value: 0.00564	0.3141[0.1908-0.4375]<br>p-value: 0
**RF**	0.6913	0.1962[0.0505-0.3419]<br>p-value: 0.00832	0.3616[0.2482-0.475]<br>p-value: 0

**GB**	0.6975	0.1871[0.0313-0.343]<br>p-value: 0.01862	0.236[0.1175-0.3544]<br>p-value: 9e-05
**LR**	0.7098	0.169[0.0478-0.2903]<br>p-value: 0.00627	0.2358[0.1425-0.329]<br>p-value: 0
**SVM**	0.7225	0.1313 [-0.0164-0.2791]<br>p-value: 0.08153	0.2884[0.177-0.3998]<br>p-value: 0
**NN**	0.7234	0.1079[0.0051-0.2108]<br>p-value: 0.03972	0.0874[-0.0015-0.1764]<br>p-value: 0.054
**CSSSNN**	0.7501	/	/

DT: decision tree; KNN: k-nearest neighbor; RF: random forest; GB: gradient boosting; LR: logistic regression; SVM: support vector machine; NN: neural network; CS+SS+NN: cost-sensitive semi-supervised neural network; NRI: net reclassification index; IDI: integrated discrimination improvement.

Table 12. Performance evaluation of models trained on down-sampling and pseudo-label dataset

Classifier	Sensitivity	Specificity	Accuracy	F1-score	NRI	IDI
**DT**	0.5472	0.7240	0.6898	0.7157	-0.0952	-0.0487
**KNN**	0.5472	0.7285	0.6934	0.7187	-0.009	0.019
**RF**	0.6038	0.7557	0.7263	0.7477	0.0537	0.0118
**GB**	0.6416	0.6878	0.6788	0.7098	0.0181	-0.0088
**LR**	0.7358	0.6923	0.7007	0.7304	0.0989	0.0819
**SVM**	0.6415	0.7285	0.7117	0.7371	-0.0106	0.116
**NN**	0.6981	0.7285	0.7190	0.7449	0.0362	0.0348
**CS+SS+NN**	0.7925	0.7059	0.7226	0.7501	/	/

DT: decision tree; KNN: k-nearest neighbor; RF: random forest; GB: gradient boosting; LR: logistic regression; SVM: support vector machine; NN: neural network; CS+SS+NN: cost-sensitive semi-supervised neural network; NRI: net reclassification index; IDI: integrated discrimination improvement.
